# Popliteal aneurysm repair using the posterior approach

**DOI:** 10.1016/j.jvscit.2022.08.026

**Published:** 2022-09-08

**Authors:** Bradley Bowles, Mark Awad, Matthew R. Smeds

**Affiliations:** Division of Vascular and Endovascular Surgery, Department of Surgery, Saint Louis University, St. Louis, MO

**Keywords:** Aneurysm, Popliteal aneurysm, Posterior, Repair, Surgery, Treatment

Popliteal artery aneurysms are the most common peripheral artery aneurysm, accounting for ≥70% of peripheral aneurysms.^1^ It has generally been accepted that the indication for repair includes a symptomatic aneurysm or an asymptomatic aneurysm ≥2 cm in a medically suitable patient.^2^ The treatment modalities can be divided into endovascular and open surgical repair with the latter subdivided into posterior and medial approaches, depending on the location and the patient’s anatomic features. Open repair has had superior patency and amputation-free survival compared with endovascular repair but with similar mortality rates and has been well tolerated with reasonable long-term results.^3^ When deciding between a posterior and medial approach, an understanding of the patient’s aneurysm characteristics is crucial. Small or fusiform aneurysms, those extending outside the popliteal fossa, and those in patients with poor outflow necessitating distal bypass will often be approached medially. Larger aneurysms with compressive symptoms and those confined to the behind the knee popliteal space will often be treated using the posterior approach.^2^

In our video, we demonstrate the treatment of a 72-year-old man with an asymptomatic 3.8-cm popliteal aneurysm using an open posterior approach and prosthetic conduit. In brief, the posterior approach was performed with the patient in the prone position via an S-shaped incision from the medial side of the thigh extending to the lateral calf with the horizontal component over the flexor crease (*A*). The upper longitudinal incision can be extended over the great saphenous vein, and the inferior incision can be extended medially over the small saphenous vein if these vessels have been targeted as conduits. An interposition bypass using either a prosthetic or saphenous vein graft has demonstrated similar patency rates and major adverse limb events in this position and improved patency compared with a medial bypass in some studies, although the data is somewhat lacking.^4^^,^^5^ The patient provided written informed consent for the report of his case details and imaging studies (available on request).

In conclusion, the posterior approach to popliteal artery aneurysms is a durable repair option for patients with pathology confined to the popliteal fossa. The procedure is well tolerated and complements endovascular and open medial approaches to this disease in properly selected patients.

**Figure:**
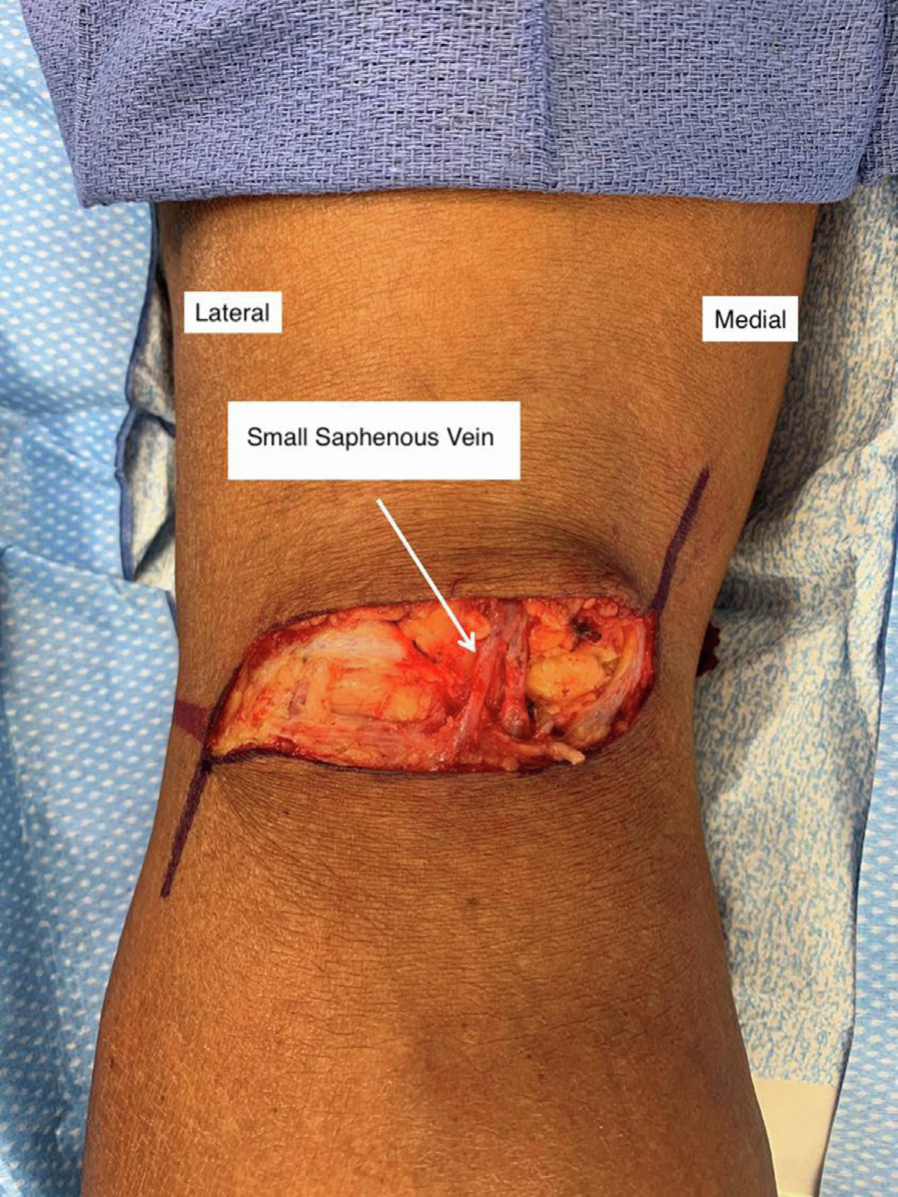
Intraoperative photograph showing incision with identification of small saphenous vein.

## Appendix

Video Popliteal aneurysm repair.
